# Hypoglycemia as a Cause of Reversible Recurrent Chorea in a Diabetic Uremic Patient: A Rare Presentation

**DOI:** 10.7759/cureus.39292

**Published:** 2023-05-21

**Authors:** Jin Zheng, Ashraf Sliem, Sergio Magana, Anil Kapoor

**Affiliations:** 1 Internal Medicine, Flushing Hospital Medical Center, New York, USA; 2 Neurology, Flushing Hospital Medical Center, New York, USA

**Keywords:** hypoglycemia-induced chorea, hypoglycemia, recurrent reversible choreiform movements, uremia, chorea

## Abstract

Chorea is characterized by involuntary, fidgety, dance-like movements caused by basal ganglia lesions. It has frequently been reported with hyperglycemia in diabetic patients, but not in association with hypoglycemia. We report on a diabetic male on hemodialysis who developed recurrent, acute, reversible choreiform movements associated with repeat episodes of hypoglycemia. Imaging was able to capture brain lesions corresponding to the acute episodes and the resolution of lesions between acute episodes.

## Introduction

Chorea is a hyperkinetic movement disorder originating from basal ganglia pathology and is clinically characterized by brief, irregular, purposeless, non-stereotypical involuntary movements [1]. Chorea can be hereditary, idiopathic, or acquired. Acquired chorea can be caused by ischemia, infection, metabolic abnormality, etc. Chorea is reported in diabetic uremic patients and might be related to uremic toxins, metabolic acidosis, and hyperglycemia. However, hypoglycemia is rarely reported to cause recurrent, reversible choreiform movement in patients with diabetic uremia. We report a diabetic patient with end-stage renal disease who developed recurrent, reversible choreiform movements caused by reversible bilateral basal ganglia lesion secondary to hypoglycemia. Imaging captured during and between episodes of chorea is illustrative of the pathophysiology associated with the choreiform movements in this patient.

## Case presentation

**Figure 1:**
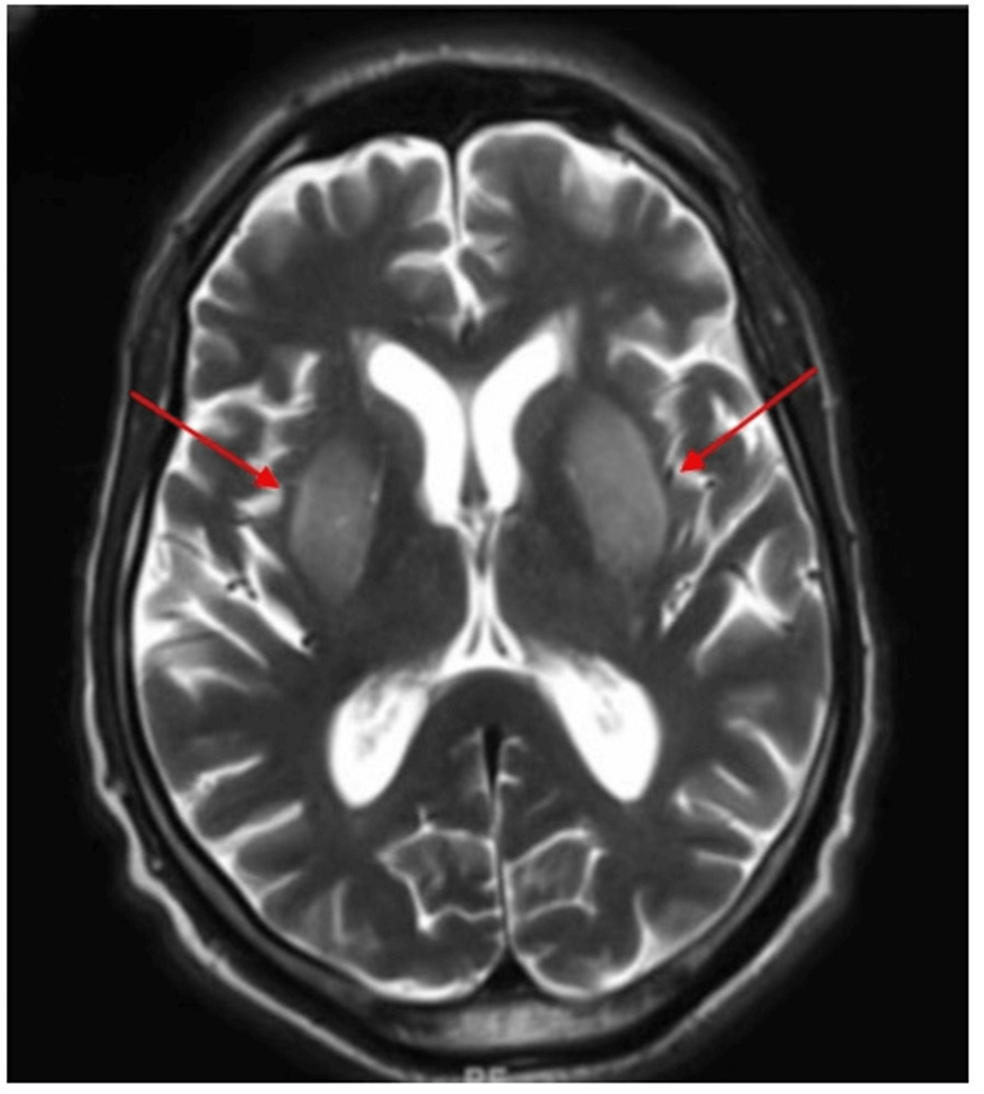
MRI brain: Day-one axial T2-weighted image shows symmetric hyperintense lesions in bilateral basal ganglia (red arrows)

**Figure 2:**
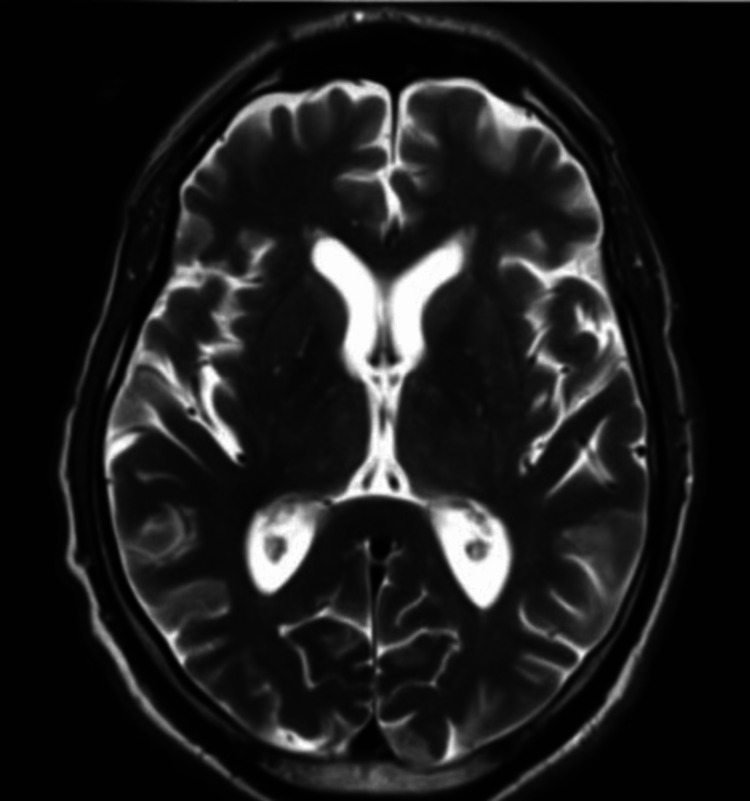
MRI brain: Day-14 axial T2-weighted image shows complete resolution of lesions noted on day one

A 71-year-old Hispanic man with a medical history of hypertension, type 2 diabetes mellitus treated with insulin, and end-stage renal disease on hemodialysis presented on two occasions with similar diffuse choreiform movements. He had no history of cerebrovascular disease or malignancy. During the first admission, his vital signs were blood pressure of 110/66 mm Hg, respiratory rate of 18 breaths/minute, pulse of 61 beats/minute, and temperature of 98.7°F. On neurologic examination, he had diffuse choreiform movements (Video 1). Cranial nerves were grossly intact. Motor and sensory examinations were otherwise unremarkable. Capillary blood glucose was 45 mg/dl, which was corrected immediately to 145 mg/dl by intravenous D50. Glycated hemoglobin (hemoglobin A1C) was 5.9%, blood urea nitrogen was 59 mg/dl, and creatinine was 9 mg/dl. According to the patient, he was compliant with the insulin regimen, which was glargine 20 units at bedtime and metformin 1000 mg daily, with blood glucose in the 160-180 mg/dl range. An extensive workup excluded autoimmune/inflammatory, infection, cerebrovascular, neoplastic, and toxic causes. T2/fluid-attenuated inversion recovery (FLAIR) brain MRI without contrast (day one) showed hyperintensity of the putamen bilaterally (Figure 1). An MRI brain done six weeks prior was unremarkable. He responded well to amantadine 100 mg once daily and aripiprazole 2.5 mg once daily (Video 2). The diabetic regimen was changed to an insulin sliding scale three times daily before meals and the patient was given diabetic education and suggested to follow up in the endocrinology clinic. A follow-up MRI of the brain (day 14) done two weeks after the onset of symptoms showed almost complete resolution of bilateral basal ganglia lesions (Figure 2). His symptoms resolved completely, so the regimen of aripiprazole and amantadine was stopped.

**Video 1:** Initial presentation with choreiform movement

**Video 2:** Resolution of symptoms after treatment

**Figure 3:**
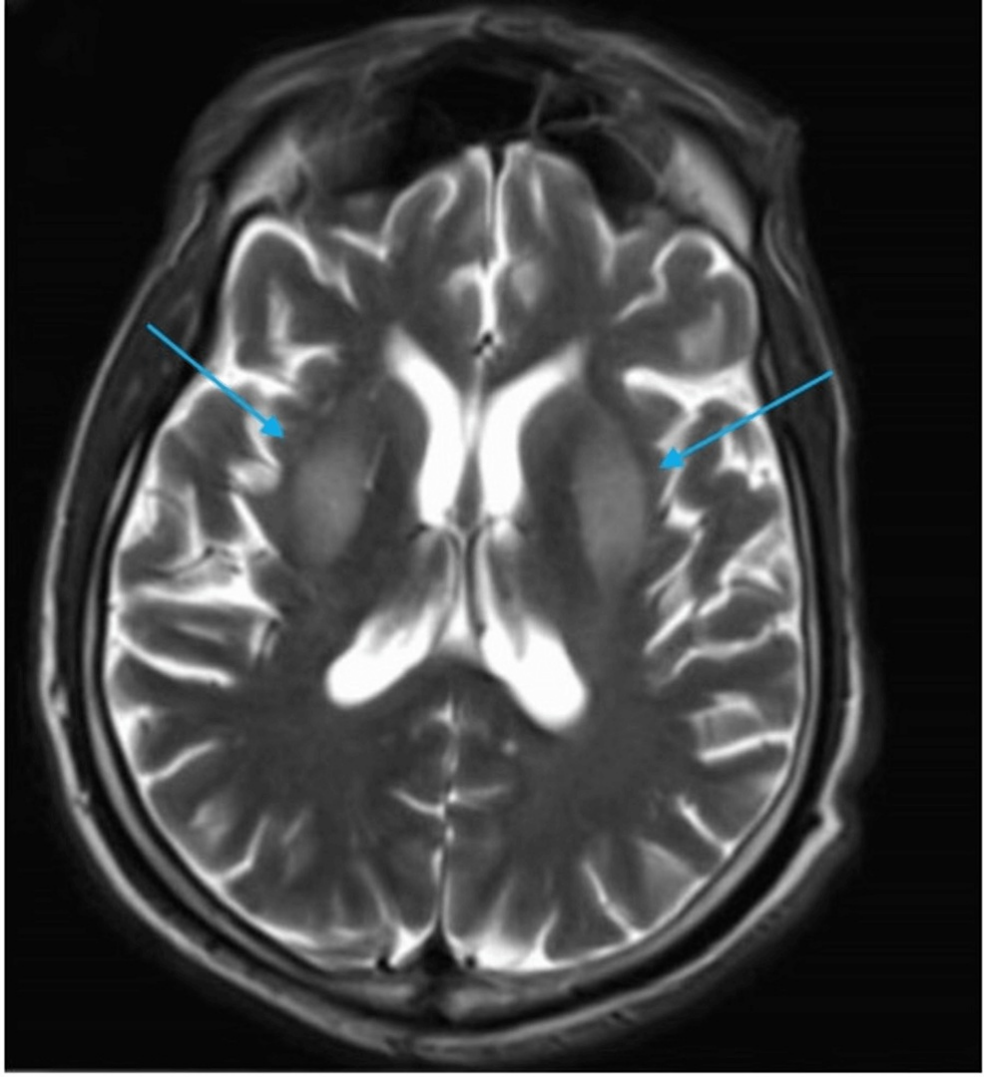
MRI brain: Day-60 axial T2-weighted image shows recurrence of symmetric hyperintense lesions in bilateral basal ganglia (blue arrows)

**Figure 4:**
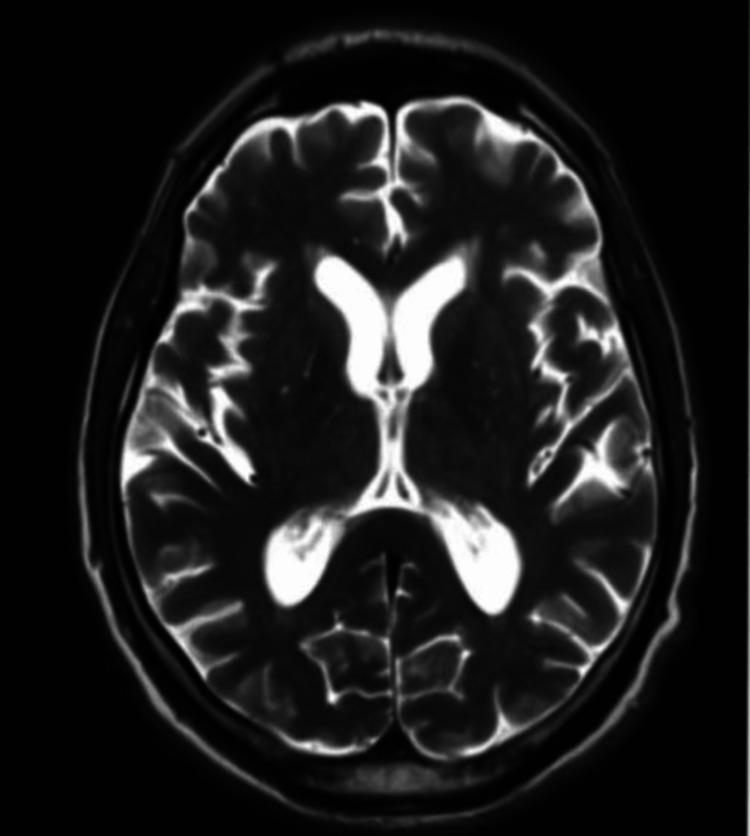
MRI brain: Day-92 axial T2-weighted image showing lesions noted on day 60 have resolved

The patient was readmitted eight weeks later with similar diffuse choreiform movements (Video 3). Capillary blood glucose was 70 mg/dL and was corrected immediately. The patient could not provide a home glucose log. MRI of the brain (day 60) again demonstrated bilateral basal ganglia T2/FLAIR abnormalities (Figure 3). He was treated with the same medications for two weeks and symptoms resolved (Video 4). MRI brain without contrast (day 92) performed after four weeks revealed a resolution of abnormalities (Figure 4).

**Video 3:** Recurrence of symptoms eight weeks after initial presentation

**Video 4:** Resolution of symptoms after treatment

## Discussion

The basal ganglia require high-energy input and blood supply for motor control. They are vulnerable to a wide range of metabolic changes, such as carbon monoxide intoxication or hypoxia, which may lead to irreversible damage. However, toxins and changes in blood glucose levels might cause reversible lesions [2]. Acute bilateral basal ganglia lesions in diabetic patients with end-stage renal disease usually present clinically as parkinsonism or dystonia, and clinical prognoses are variable. Interestingly, rare cases present as choreiform movement; these show a more favorable prognosis [3]. Hyperglycemia has been recognized as a trigger leading to basal ganglia lesions in patients with diabetic uremia. However, hypoglycemia has rarely been reported as a possible trigger [2,4]. To our knowledge, this is the first report of recurrent, reversible choreiform movements caused by reversible bilateral basal ganglia lesions in a diabetic uremic patient secondary to hypoglycemia. The pathogenesis of bilateral basal ganglia lesions in a patient with diabetic uremia with abnormal blood glucose levels is still unclear. The possible pathogenic mechanisms underlying are multifactorial, including sudden blood glucose level changes that can increase the blood-brain barrier permeability or uremic toxins that may lead to basal ganglia lesions based on cerebrovascular endothelial dysfunction, which can trigger oxidative stress and damage related to brain tissue [5,6]. Furthermore, some cases report bilateral basal ganglia lesions induced by hypoglycemia to have low apparent diffusion coefficient (ADC) values corresponding to cytotoxic edema or neuronal necrosis [7]. Our case also shows low ADC values, which may relate to a more favorable prognosis. In the future, early diffuse weighted imaging (DWI)/ADC imaging may be more instructive than the ambiguous duration of hypoglycemia for predicting clinical outcomes [2,8]. Also, immediate treatment of abnormal blood glucose may prevent irreversible brain damage.

## Conclusions

Hypoglycemia in diabetic uremic patients may lead to bilateral basal ganglia lesions. Providers should stabilize glucose levels in patients with diabetic uremia because sudden changes in blood glucose can lead to acute bilateral basal ganglia lesions. Also, patients presenting with choreiform movement may show a more favorable prognosis. Thus, the provider must be cognizant of this entity and treat blood glucose abnormalities immediately in a patient with diabetic uremia presenting with choreiform movements to prevent irreversible pathologic changes. Only symptomatic treatment is needed for patients if blood glucose levels are correct immediately and the symptoms persist. Also, early DWI/ADC imaging may be an instructive tool for predicting clinical outcomes.
